# *Edgeworthia gardneri* (Wall.) Meisn. extract protects against myocardial infarction by inhibiting NF-κB-and MAPK-mediated endothelial inflammation

**DOI:** 10.3389/fcvm.2022.1013013

**Published:** 2022-12-20

**Authors:** Dan Wei, Le Tang, Lingqing Su, Sufen Zeng, Ajdora Telushi, Xiaoya Lang, Yanli Zhang, Manman Qin, Liang Qiu, Chao Zhong, Jun Yu

**Affiliations:** ^1^Center for Translational Medicine, Jiangxi University of Chinese Medicine, Nanchang, Jiangxi, China; ^2^Department of Cardiovascular Sciences and Center for Metabolic Disease Research, Lewis Katz School of Medicine, Temple University, Philadelphia, PA, United States; ^3^The National Pharmaceutical Engineering Center for Solid Preparation in Chinese Herbal Medicine, Jiangxi University of Chinese Medicine, Nanchang, Jiangxi, China

**Keywords:** *Edgeworthia gardneri* (Wall.) Meisn., myocardial infarction, inflammation, endothelial cells, NF-κB, MAPK

## Abstract

**Background:**

Experimental and clinical evidence has demonstrated a pivotal role of inflammation in the pathogenesis of ischemic heart disease, and targeting inflammation has been shown to provide clinical benefits for patients with coronary disease. Endothelial cells constitute the majority of non-cardiomyocytes in the heart. Endothelial pro-inflammatory activation is recognized as a critical component in the pathophysiology of cardiovascular disease. The dried flowers of *Edgeworthia gardneri* (Wall.) Meisn. (EG) have been widely used as Tibetan folk medicine to ameliorate a range of metabolic disorders, such as diabetes mellitus, hyperlipidemia, hypertension, and obesity. However, its role in modulating endothelial inflammation and ischemic heart disease has not been evaluated.

**Methods and results:**

Herein, using a preclinical rat model of coronary artery ligation-induced myocardial infarction (MI), we demonstrated that systemic administration of EG extract (EEEG) attenuated ischemic cardiac injury. EEEG reduced myocardial infarct size, improved cardiac function, and ameliorated adverse cardiac remodeling. Moreover, the cardioprotective effects of EEEG were associated with decreased MI-induced myocardial inflammation. Consistent with the anti-inflammatory role of EEEG *in vivo*, EEEG attenuated TNF-α-stimulated human umbilical vein endothelial cells (HUVECs) activation and monocyte-endothelial cell firm adhesion *in vitro*. Mechanistically, our data showed that EEEG’s mode of action suppresses the activation of NF-κB, ERK, and p38 MAPK signaling pathways in ECs. Importantly, we demonstrated that EEEG inhibits endothelial inflammation in an NF-κB- and p38 MAPK-dependent manner using pharmacological inhibitors.

**Conclusion:**

Collectively, this study identified EG as a potential therapeutic agent in attenuating endothelial inflammation and managing ischemic cardiovascular disease.

## Introduction

Myocardial infarction (MI) refers to an event of a heart attack characterized by inhibiting blood flow to the heart that irreversibly causes cardiac injury and impairs cardiac function, eventually leading to the pathogenesis and progression of heart failure (1, 2). It has been reported that there are currently about 126.5 million cases of ischemic heart disease (IHD), with a concomitant of over 9 million deaths per year in the world, making IHD a leading cause of morbidity and mortality worldwide (3, 4). Over the past years, despite significant advances in the clinical practice for preventing and treating IHD, it has caused a substantial burden on healthcare resources worldwide (5, 6). Therefore, much work remains to be done to seek new effective strategies for managing IHD.

Inflammation is implicated in MI and plays a critical role in the onset and progression of IHD (7). Upon cardiac injury following MI, the release of danger signals activates the innate immune signaling and triggers an overwhelming inflammatory response to clear the necrotic cardiomyocytes from the injured heart (7). This pro-inflammatory state is a finely orchestrated process followed by an anti-inflammatory state to promote cardiac repair (7, 8). Notably, a proper physiologic equilibrium between these pro-inflammatory and anti-inflammatory phases contributes to optimal post-infarct healing and cardiac remodeling (8). Excessive and prolonged inflammation may impair wound healing and cause cell loss and cardiac dysfunction, leading to sustained cardiac injury and adverse remodeling events (8). Anti-inflammatory therapies among patients with recent MI or chronic coronary disease have resulted in a lower risk of ischemic cardiovascular events (9, 10), indicating that controlled inflammation may be a fundamental determinant for favorable cardiovascular outcomes. In the heart, endothelial cells (ECs) are recognized to be the most abundant non-cardiomyocytes. The endothelial pro-inflammatory activation plays a critical role in the recruitment of leukocytes into the infarcted area and the subsequent inflammatory cascades after MI (8, 11). Thus, manipulating endothelial inflammation is a potential therapeutic strategy for attenuating cardiac inflammatory damage during MI.

Nuclear factor κB (NF-κB) and mitogen-activated protein kinases (MAPKs), including p38 MAPK, extracellular signal-regulated protein kinases1/2 (ERK1/2), and c-Jun N-terminal kinase (JNK) are pivotal intracellular signaling molecules. They jointly regulate many physiological and pathological processes, including inflammation (8, 12, 13). In the context of MI, intracellular signaling converges on the activation of NF-κB and MAPK pathways to induce cardiac inflammatory responses (8). Remarkably, NF-κB and MAPK inhibition have been demonstrated to be beneficial for MI-induced injury with the manifestation of attenuated infarct size, reduced inflammatory responses, and improved cardiac function (14, 15). Therefore, targeted intervention in NF-κB and MAPK signaling pathways provides an attractive strategy for protecting against MI injury via modulating immune responses.

The dried flowers of *Edgeworthia gardneri* (Wall.) Meisn. (EG), also known as “Lv Luo Hua” in China, have long been widely used as traditional Tibetan medicine to prepare a herbal beverage to treat and prevent a range of diseases such as diabetes mellitus, hyperlipidemia, hypertension, and obesity (16–19). In addition, the active components of EG, including quercetin (20), tiliroside (21), umbelliferone (22), and pentadecanoic acid (22), have been identified and shown to possess excellent pharmacological properties such as islet protection, α-glucosidase inhibition and PPARγ/β activation. However, the precise role of EG in IHD remains elusive. Thus, in the present study, we aim to investigate whether EG affects the pathogenesis of MI and, if so, to reveal the potential mechanisms using a rat model of acute ischemic myocardial injury.

## Materials and methods

### Chemicals and reagents

Ethanol (64-17-5) and petroleum ether (8032-32-4) were purchased from Xilong Science (Guangzhou, China). AB-8 Macroporous Adsorptive Resin was provided by Baoen Chemical (BE1003, Hebei, China). Sodium carboxymethyl cellulose (CMC-Na) was obtained from Zhanyun Chemical (9004-32-4, Shanghai, China). Dimethyl sulfoxide (DMSO) was purchased from Solarbio (D8370, Beijing, China). 2,3,5-triphenyl tetrazolium chloride (TTC) was obtained from Solarbio (T8170, Beijing, China). Xylene was provided by Damao Chemical (3833, Tianjin, China). 10x Zinc Fixative solution was purchased from BD Biosciences (552658, Franklin Lakes, NJ, USA). Tumor necrosis factor-α (TNF-α) was purchased from PeproTech (300-01A, New Jersey, USA). Vascular endothelial growth factor-A (VEGF-A) was obtained from Lonza (CC-4114A, Basel, Switzerland). p65 (6956), p-p65 (3033), p38 MAPK (8690), p-p38 MAPK (9216), ERK (4696), and p-ERK (9101) antibodies were purchased from Cell Signaling Technology (Danvers, MA, USA). CD45 (ab10558), CD68 (ab125212), and CD31 (ab64543) antibodies were purchased from Abcam (Cambridge, UK). Goat anti-rabbit IgG (I31203) and goat anti-mouse IgG (I31107) were purchased from TransGen Biotech (Beijing, China). Biotin-conjugated goat anti-rabbit IgG (H + L) (ABM120002-100) and goat anti-mouse IgG (H + L) (ABM120001-100) was purchased from Embime Biology (Beijing, China). Streptavidin-Horseradish Peroxidase was purchased from Lianke Biotech (SH001, Hangzhou, China). 3,3′-diaminobenzidine was purchased from Invitrogen (750118, Carlsbad, CA, USA). Masson’s trichrome Staining Kit was purchased from Sigma-Aldrich (1004850001, St. Louis, MO, USA). Hematoxylin (51275) and eosin (E4009) were purchased from Sigma-Aldrich (St. Louis, MO, USA). Trizol reagent was purchased from Invitrogen (15596-018, Carlsbad, CA, USA). High-Capacity cDNA Reverse Transcription Kit (RR047A) and SYBR Green PCR Master Mix (RR820A) were purchased from Takara (Shiga, Japan). EGM-2 endothelial growth medium was purchased from Lonza (CC-3156 & CC-4176, Basel, Switzerland). MTT was purchased from Sigma-Aldrich (M5655, St. Louis, MO, USA). RIPA buffer was purchased from KeyGen (KGP703-100, Jiangsu, China). Protease and phosphatase inhibitors were purchased from Vazyme (E312-01, Jiangsu, China). NF-κB inhibitor BAY11-7082 (B5556), ERK inhibitor SD98059 (P215), and p38 MAPK inhibitor SB203580 (S8307) were purchased from Sigma-Aldrich (St. Louis, MO, USA).

### Plant material

The medicinal plant material was purchased from Zangxi Tang Company (Tibet, China), and its sample was authenticated as dried flowers of *Edgeworthia gardneri* (Wall.) Meisn. by Dr. Guoyue Zhong (Jiangxi University of Chinese Medicine). A voucher sample (GH827) was deposited at Jiangxi University of Chinese Medicine.

### Preparation of EG extract

EG (2.7 kg) was initially soaked in 70% ethanol (Xilong Science) and then decocted to an extract solution, followed by rotary evaporation of the extract solution under reduced pressure. Subsequently, the concentrated extracts were further extracted with petroleum ether (Xilong Science), and then the petroleum ether extract and the residue fraction were obtained, respectively. The residue fraction was concentrated under reduced pressure and then separated by macroporous resin column chromatography (Baoen Chemical) and eluted with a gradient system of water-ethanol (100:0 and 70:30). The resultant 30% ethanol fraction of EG extract (EEEG) was harvested and evaporated with a rotary evaporator under reduced pressure followed by vacuum freeze drying. Before *in vitro* studies, EEEG was dissolved in DMSO (Solarbio) to get a stock solution followed by further dilution to a final concentration with the cell culture medium. For animal experiments, EEEG was dissolved in 0.1% CMC-Na (Zhanyun Chemical).

### Ultra performance liquid chromatography (UPLC)-mass spectrometry (MS) analysis

Identification and characterization of chemical constituents of EEEG were performed using UPLC-Q-TOF-MS. 50 mg EEEG was dissolved in 10 ml water with ultrasound followed by centrifugation at 12,000 rpm for 15 min at 4°C, the supernatant was filtered and injected for further UHPLC-Q-TOF-MS analysis. The reference compound tiliroside (20316-62-5, Desite Biotechnology, Sichuan, China) with purity >98% was also used for this analysis. The separation was performed on a Nexera X2LC-30A system (Shimazdu Corp., Japan) using an Acquity UPLC HSS T3 column (100 × 2.1 mm, i.d., 1.8 μm, Waters, USA). Chromatographic separation conditions were as follows: the sample injection volume was 5 μl, the column temperature was 40°C, and the flow rate was 0.3 ml/min. The mobile phase was composed of 0.1% formic acid in water (A) and acetonitrile (B), and the gradient elution procedure was as follows: 0–3 min, 2–10% B; 3–12 min, 10–17.5% B; 12–20 min, 17.5–18% B; 20–27 min, 18–30% B; 27–34 min, 30–60% B; 34–39 min, 60–95% B; 39–42 min, 95% B; 42–42.01 min, 95–2% B; 42.01–45 min, 2% B. The Q-TOF-MS analysis was performed using TripleTOF 5600 mass spectrometer (AB SCIEX, Framingham, MA, USA) in positive electrospray ionization mode. The scan range was m/z 50–1,250, with a spray voltage of 4,500 V, a nebulizing gas temperature of 500°C, a curtain gas pressure of 25 psi, a nebulizing gas pressure of 50 psi, an auxiliary gas pressure of 50 psi, a declustering potential of 100 V, and a collision energy of 30 eV. Data analysis was performed using Analyst TF 1.6 software and PeakView software (AB SCIEX, Framingham, MA, USA). Chemical constituents of EEEG were determined according to the retention time and MS fragmentations. The total ion chromatograms (TICs) of EEEG and the reference compound by UPLC-Q-TOF-MS, data analysis of UPLC-Q-TOF-MS and the chemical structures of identified constituents in EEEG were shown in Supplementary Figures 1–3 and Supplementary Table 1.

### Animals

Adult male Sprague-Dawley rats (200 ± 20 g) were obtained from Silaike (SCXK-2013-0004, Hunan, China) and housed in the Experimental Animal Center of Jiangxi University of Chinese Medicine (license number: SYXK-2017-0004). All studies were performed according to the guidelines approved by the Institutional Animal Care and Use Committee of Jiangxi University of Chinese Medicine. All rats were maintained in controlled conditions with free access to food and water (temperature: 23 ± 2°C, humidity: 60 ± 5%, and 12 h/12 h light/dark cycle). The rats used in this study were randomly assigned to three groups (*n* = 8 per group): sham operation group, MI surgery group, and MI + EEEG group. EEEG (10 g/kg) was orally administered by gastric gavage every other day for rats in the MI + EEEG group after MI surgery. The gavage dose in the present study was determined according to the equivalent patient dose. The rats in the sham group and the MI group received 0.1% CMC-Na (Zhanyun Chemical), and all rats in this study were treated for 4 weeks.

### Induction of MI

Myocardial infarction was induced by permanent ligation of the left anterior descending (LAD) coronary artery. Briefly, rats were initially anesthetized with sodium pentobarbital (60 mg/kg by intraperitoneal injection). Subsequently, the left thoracotomy was performed, the pericardium was excised, and the LAD artery was exposed. A 6/0 silk suture was then placed around the proximal LAD coronary artery 2–3 mm from its origin and tightly tied to cause occlusion of the LAD coronary artery. Next, the heart was immediately put back into the chest cavity, followed by manual air evacuation and closure of muscle and the skin. After fully recovering from anesthesia, the rats were placed back into the housing facility. The same procedure was performed for sham-operated rats without ligating the LAD coronary artery. At 4 weeks after surgery, rats were sacrificed, and tissues were collected for subsequent experiments.

### Echocardiography

Rats were mildly anesthetized using sodium pentobarbital. After removing the hairs on the chest with depilatory paste, the rat was placed on an experimental animal plate with a heating function. Then the electrocardiogram metal electrode on the animal plate was coated with a conductive paste. Next, the chest of each rat was covered with an ultrasonic coupling agent. M-mode echocardiography was performed to measure cardiac function using a Vevo3100 instrument with a 38 MHz transducer (Visual Sonics). The cardiac parameters including left ventricular end-systolic diameter (LVIDs), left ventricular end-diastolic diameter (LVIDd), anterior wall thickness (AWT), the ratio of mitral peak velocity during early diastole to atrial contraction (E/A ratio), ejection fraction (EF), and fractional shortening (FS) were measured and calculated.

### Measurement of myocardial infarct size

Briefly, rat hearts were harvested and cleared of blood with chilled PBS. Next, the rat hearts were frozen at −80°C for 10 min and then sliced transversally from apex to base at 3–5 mm thick, followed by the incubation of 1% TTC solution (Solarbio) for 30 min at 37°C in the dark. Subsequently, the slices were treated with 1x Zinc Fixative solution (BD Biosciences) for 24 h. Mark the infarct size and calculate using ImageJ (National Institutes of Health, Bethesda, MD, USA). Express the infarct size as a ratio of the infarct area (the unstained necrotic area) vs. the total left ventricle (LV) area.

### Histology and immunohistochemistry

Heart specimens were rinsed with cold PBS, fixed in 1x Zinc Fixative solution (BD Biosciences), embedded in paraffin, and cut into 5 μm thick sections. These sections were then incubated with a graded ethanol series for deparaffinization and hydration. Heart sections were stained with Masson’s trichrome (Sigma-Aldrich) to visualize myocardial fibrosis, hematoxylin, and eosin (H&E) (Sigma-Aldrich) to show the heart structure, as previously described (23, 24). For immunohistochemistry, sections were incubated with anti-CD45 (Abcam), anti-CD68 (Abcam), and anti-CD31 (Abcam) primary antibodies, followed by biotin-conjugated secondary antibodies (Embime). Next, sections were treated using Streptavidin-Horseradish Peroxidase (Lianke) and 3,3′-diaminobenzidine (Invitrogen), followed by counter-staining with hematoxylin (Sigma-Aldrich).

### Quantitative real-time PCR (qRT-PCR)

Total RNA from rat hearts and cultured cells was isolated using Trizol reagent (Invitrogen), and reverse transcription was performed using the High-Capacity cDNA Reverse Transcription Kit (Takara). The resulting cDNA was amplified for 35 cycles using SYBR Green PCR Master Mix (Takara) by the ABI 7500 Real-Time PCR System. Data were normalized by *Gapdh* software. The sequences of primers used in this study are shown in Supplementary Table 2.

### Culture of human umbilical vein endothelial cells (HUVECs)

HUVECs were obtained from the Vascular Biology and Therapeutics Program of Yale University and cultured in an EGM-2 endothelial growth medium (Lonza). Cells were incubated at 37°C in a humidified atmosphere containing 5% CO_2_. Passage 4–10 was used for experiments.

### Cell viability and proliferation assay

Cell viability was assessed by MTT assay. Briefly, HUVECs (1.2 × 10^4^ cells/well) were cultured in 96-well plates. Upon growth to sub-confluence, the cells were treated with or without different concentrations of EEEG for 24 h. After washing with PBS, MTT (5 mg/ml) (Sigma-Aldrich) was used to treat cells at 37°C for 3 h. Subsequently, the medium was discarded, and 100 μl of DMSO (Solarbio) was added into each well to dissolve formazan blue generated within the cells. The optical density was measured at 490 nm using a microplate reader (Bio-Rad). Cell viability in each well was expressed as a percentage of the vehicle control. Cell proliferation assay was performed by direct cell counting. HUVECs were seeded at 1 × 10^5^ cells/well in 24-well plates and treated with EEEG (500 μg/mL) for indicated time points. After incubation, cells were harvested and counted using a hemocytometer.

### Cell adhesion assay

HUVECs (1 × 10^5^ cells/well) were seeded in 24-well plates and pretreated with EEEG (500 μg/mL) for 24 h. When the cells reached confluence, cells were stimulated with TNF-α (10 ng/mL) (PeproTech) for 4 h. Subsequently, the medium was removed, and Dil-labeled human acute monocytic leukemia THP-1 cells (5 × 10^4^ cell/well) were added to HUVECs. Cells were co-cultured for another 2 h in a 5% CO_2_ incubator at 37°C. Next, wash with PBS to remove the non-adherent cells. THP-1 monocyte adhesion was quantified in five random fields per well by a fluorescence microscope (Nikon).

### Western blot

Cells were homogenized in cold RIPA buffer (KeyGen) supplemented with protease and phosphatase inhibitors (Vazyme). Total protein extracts (30 μg) were separated by 10% SDS-polyacrylamide gel electrophoresis and transferred to nitrocellulose membranes (Millipore). Five percentage BSA in Tris-buffered saline/Tween-20 (TBST) was used to block membranes for 1 h. The primary antibody incubation was performed overnight at 4°C followed by secondary antibody treatment for 1 h at room temperature. Signals were visualized using ODYSSEY Infrared Imaging System (LI-COR). β-actin and Hsp90 were used as loading controls. Signal intensities were quantified using Image Studio software (LI-COR).

### Statistical analysis

All data are presented as mean ± standard errors of the means (SEM). Statistical analysis was performed using Prism 8 software (GraphPad, CA, USA). The statistical significance of differences was evaluated using a 2-tailed Student’s *t*-test for comparisons between two groups and one-way ANOVA followed by the Tamhane T2 test for multiple groups comparisons. Differences with a *p*-value < 0.05 were considered to denote statistical significance.

## Results

### EEEG attenuates MI-induced infarction and improves cardiac function *in vivo*

**FIGURE 1:**
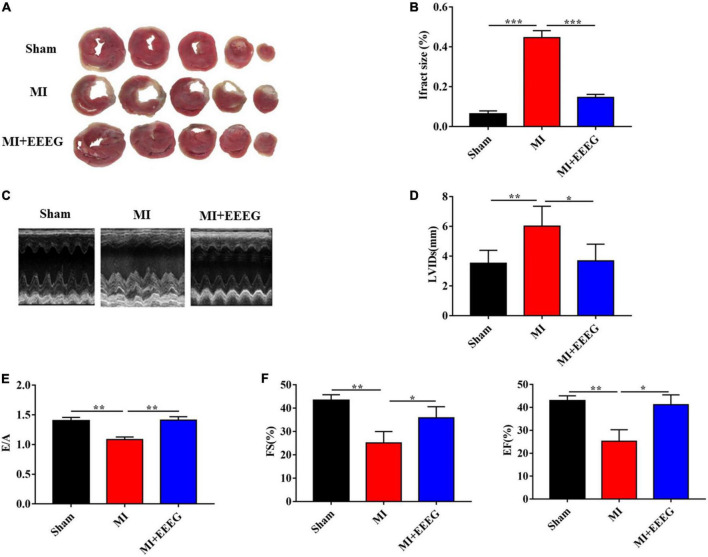
EG extract (EEEG) reduces infarct area and protects against cardiac dysfunction after MI. **(A)** Representative TTC staining of cardiac tissue obtained from vehicle and EEEG (10 g/kg) treated rats at 4 weeks after MI or sham operation. **(B)** Quantitative analysis of TTC-stained infarct area 4 weeks after MI or sham operation in vehicle and EEEG (10 g/kg) treated rats (*n* = 8). **(C)** Representative M-mode tracing for vehicle and EEEG (10 g/kg) treated rats at 4 weeks after MI or sham operation are shown. **(D–F)** Echocardiographic analysis of left ventricular end-systolic diameter **(D)**, the ratio of mitral peak velocity during early diastole to atrial contraction **(E)**, ejection fraction (**F**, left panel), and fractional shortening (**F**, right panel) at 4 weeks after MI or sham operation in vehicle and EEEG (10 g/kg) treated rats (*n* = 8). Data are mean ± SEM. *P < 0.05, **P < 0.01, ***P < 0.001.

To determine whether EEEG affects ischemic myocardial injury, we performed permanent LAD ligation to induce MI in rats, followed by oral administration of EEEG or vehicle. First, we measured infarct size using TTC staining to examine MI-induced myocardial necrosis. As expected, the infarct size was markedly increased in vehicle-treated rats that underwent 4 weeks of MI compared with sham-operated controls (Figures 1A, B). The hearts of EEEG-treated rats showed significantly decreased infarct size compared to vehicle controls 4 weeks after MI (Figures 1A, B). Next, we performed M-mode echocardiography to investigate the influence of EEEG on cardiac function upon MI injury. As demonstrated by representative echocardiographic images (Figure 1C) and analyses (Figures 1D–F), EEEG exhibited protective effects on ventricular functions as shown by reduced LVIDs, higher E/A ratio, improved EF and FS compared to vehicle controls. Furthermore, EEEG treatment decreased heart weight-to-femur length and heart-to-body weight ratios, indicating their improved cardiac hypertrophy and function at 4 weeks post-MI compared to vehicle-treated rats (Supplementary Figure 4). These data suggest that EEEG treatment protects against myocardial damage and cardiac dysfunction resulting from MI.

### EEEG alleviates MI-induced adverse cardiac remodeling *in vivo*

**FIGURE 2:**
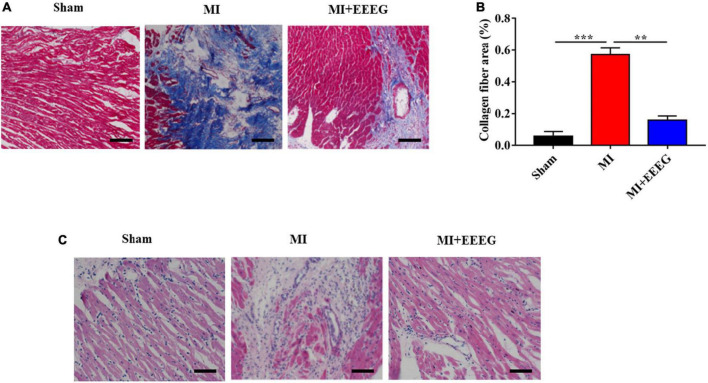
EG extract (EEEG) alleviates fibrotic remodeling and histopathologic changes of cardiac tissue in response to MI. **(A)** Representative Masson’s trichrome-stained sections of vehicle and EEEG (10 g/kg) treated rat hearts 4 weeks after MI or sham operation (scale bar, 50 μm). **(B)** Masson’s trichrome staining quantified the total cardiac fibrotic area using sections of vehicle and EEEG (10 g/kg) treated rat hearts at 4 weeks after MI or sham operation (*n* = 8). **(C)** Representative H&E staining of heart tissue 4 weeks after MI or sham operation from rats treated with vehicle or EEEG (10 g/kg) (scale bar, 50 μm). Data are mean ± SEM. **P < 0.01, ***P < 0.001.

To evaluate the effects of EEEG on myocardial fibrosis remodeling after MI, Masson’s trichrome staining was used to examine collagen deposition in cardiac tissues. As expected, MI surgery resulted in a considerable increase in collagen deposition and the extent of cardiac fibrosis as compared with sham-operated rats (Figures 2A, B). EEEG treatment decreased collagen deposition in the infarcted hearts compared to vehicle controls at 4 weeks post-MI (Figures 2A, B). In addition, H&E staining was performed to examine the pathological morphologies of heart tissue. As shown in Figure 2C, Myocardial cells are arranged orderly without inflammatory cell infiltration observed in sham-operated hearts. In contrast, disorderly arranged myocardial fibers with large amounts of fibrous tissue hyperplasia in the intercellular space and massive inflammatory cell infiltration were noticed in hearts that underwent 4 weeks of MI. These cardiac pathological changes were notably counteracted by EEEG treatment (Figure 2C). Collectively, these results demonstrate that EEEG administration attenuates adverse cardiac remodeling in response to MI injury.

### EEEG reduces inflammation in the heart after MI

**FIGURE 3:**
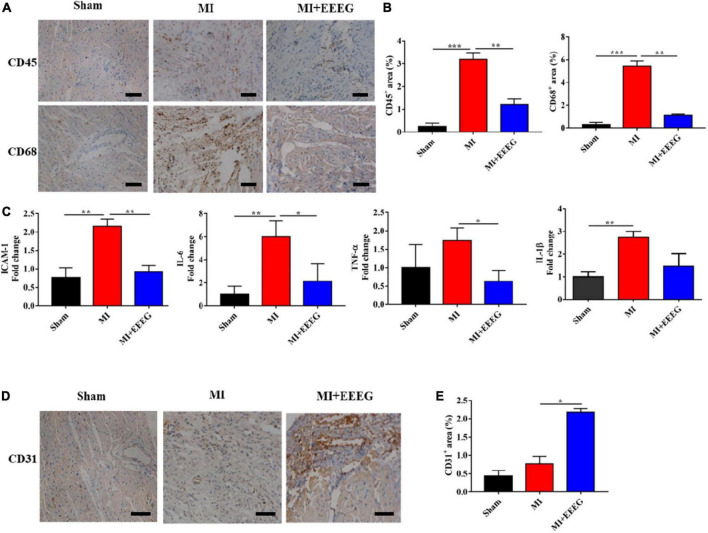
EG extract (EEEG) attenuates myocardial inflammation after MI. **(A)** Representative immunohistochemical staining of CD45 and CD68 in the heart tissue from vehicle and EEEG (10 g/kg) treated rats at 4 weeks after MI or sham operation (scale bar, 50 μm). **(B)** According to immunohistochemical staining, CD45^+^ and CD68^+^ positive areas were quantified using hearts 4 weeks after MI or sham operation from vehicle and EEEG (10 g/kg) treated rats (*n* = 8). **(C)** qRT-PCR analysis of *Icam-1*, *Il-6*, *Tnf*-α, and *Il-1*β was performed using mRNA isolated from vehicle and EEEG (10 g/kg) treated rat hearts at 4 weeks after MI or sham operation (*n* = 8). **(D)** Immunohistochemical analyses of CD31 in the cardiac tissue at 4 weeks after MI or sham operation (scale bar, 50 μm). **(E)** Quantification of CD31^+^ positive area in vehicle and EEEG (10 g/kg) treated rat hearts 4 weeks after MI or sham operation (*n* = 8). Data are mean ± SEM. *P < 0.05, **P < 0.01, ***P < 0.001.

MI injury leads to the activation of innate immune signaling and the induction of a heightened inflammatory response. The impaired suppression of post-infarction inflammation has been reported to result in adverse cardiac remodeling associated with major adverse clinical events (7, 8). To evaluate the effects of EEEG on cardiac inflammatory response following MI, rats subjected to either MI or sham surgery were treated with EEEG or vehicle, and immunohistochemical staining was used to examine the infiltration of CD45^+^ leukocytes and CD68^+^ macrophages in the myocardium. LAD ligation markedly upregulated the number of infiltrated CD45^+^ leukocytes and CD68^+^ macrophages in hearts obtained from vehicle-treated MI rats compared to those from sham-operated rats (Figures 3A, B). However, the infiltration of these immune cells was significantly reduced by EEEG administration in comparison with the vehicle controls at 4 weeks after MI (Figures 3A, B). The extent of cardiac inflammation was also investigated by examining the expression of pro-inflammatory mediators. Quantitative RT-PCR analysis showed that MI surgery significantly increased the expression of cell adhesion molecule *Icam-1* and pro-inflammatory cytokines *Il-6*, *Tnf*-α, and *Il-1*β. These effects were primarily reversed by EEEG treatment (Figure 3C). Taken together, these findings demonstrate that EEEG exerts an anti-inflammatory property which may serve as the underlying mechanism to protect against MI injury. In addition, we also observed that EEEG treatment induced a higher percentage of ECs in the infarcted hearts, as indicated by immunohistochemical analysis of CD31 positivity 4 weeks after MI. This exciting finding suggested that EEEG promoted neovascularization and wound healing (Figures 3D, E), which is worth further study.

### EEEG attenuates endothelial pro-inflammatory activation through modulation of NF-κB and MAPK signaling

**FIGURE 4:**
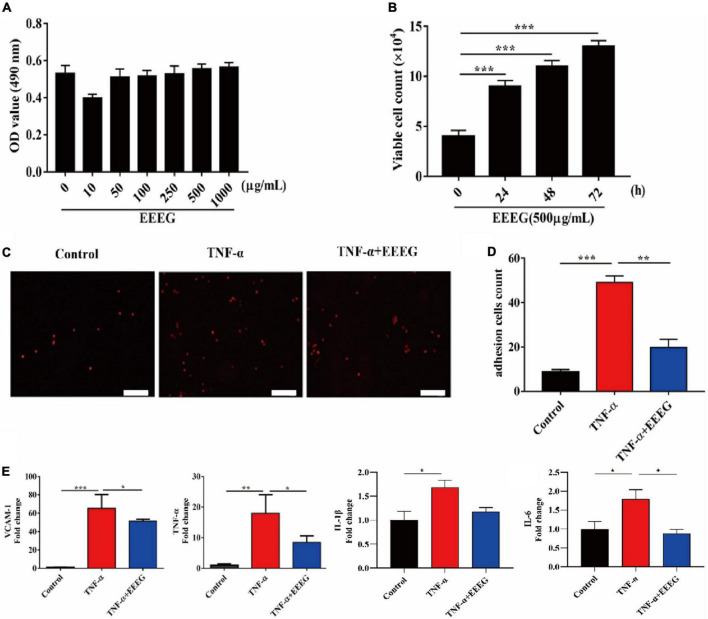
EG extract (EEEG) suppresses pro-inflammatory activation of HUVECs. **(A)** HUVECs were treated with different doses of EEEG (0, 10, 50, 100, 250, 500, and 1,000 μg/ml) for 24 h, and cell viability was determined by MTT assay (*n* = 5). **(B)** HUVECs were incubated with EEEG (500 μg/mL) for 0, 24, 48, and 72 h, and then cell proliferation was examined by direct cell counting (*n* = 3). **(C,D)** Representative images of adhesion of fluorescence-labeled THP-1 cells to HUVECs pretreated with or without EEEG (500 μg/mL) for 24 h followed by stimulation with or without TNF-α (10 ng/ml) for 4 h **(C)** (scale bar, 50 μm), and quantification of the adherent THP-1 cells (*n* = 3) **(D)**. **(E)** Gene expression levels of *Vcam-1*, *Tnf*-α, *Il-1*β, and *Il-6* in HUVECs pretreated with or without EEEG (500 μg/mL) for 24 h followed by stimulation with or without TNF-α (10 ng/ml) for 4 h (*n* = 3). Data are mean ± SEM. *P < 0.05, **P < 0.01, ***P < 0.001.

EC activation and inflammation are critical elements in the pathogenesis of ischemic heart failure (25). Upon stimulation by pathogenic mediators, endothelial pro-inflammatory cascades are triggered, including intensified adhesive interaction with circulating leukocytes and upregulated production of pro-inflammatory cytokines, which may contribute to ischemic myocardial injury when dysregulated (8). We then tested whether the anti-inflammatory effect of EEEG *in vivo* could be attributed to the attenuation of endothelial pro-inflammatory activation. Thus, TNF-α-stimulated HUVECs were used as a model of endothelial inflammation. First, we examined HUVEC viability by using an MTT assay. Our result showed that EEEG treatment alone did not affect the viability of HUVECs (Figure 4A). Interestingly, a time-dependent and dose-dependent increase in cell proliferation was observed in EEEG-treated HUVECs (Figure 4B and Supplementary Figure 5). Monocyte adhesion mediated by increased expression of endothelial adhesion molecules is a hallmark of EC pro-inflammatory activation (8). We, therefore, investigated whether EEEG could suppress THP-1 cell adhesion to TNF-α-stimulated HUVECs. As shown in Figures 4C, D, after TNF-α stimulation, monocytes’ firm adhesion was significantly increased as compared with negative controls. However, pretreatment of HUVECs with EEEG marked a decrease in monocyte adhesion to TNF-α-activated HUVECs (Figures 4C, D). Since EEEG could suppress TNF-α-induced cell adhesion, we analyzed whether EEEG could inhibit the expression level of adhesion molecules and pro-inflammatory cytokines in HUVECs in response to TNF-α. As expected, TNF-α stimulation significantly upregulated the gene expression of adhesion molecule *Vcam-1* as well as pro-inflammatory cytokines *Tnf*-α, *Il-1*β, and *Il-6* (Figure 4E). EEEG markedly attenuated the effect of TNF-α-induced expression of *Vcam-1*, *Tnf*-α, *Il-6*, and to a lesser extent *Il-1*β in HUVECs (Figure 4E).

**FIGURE 5:**
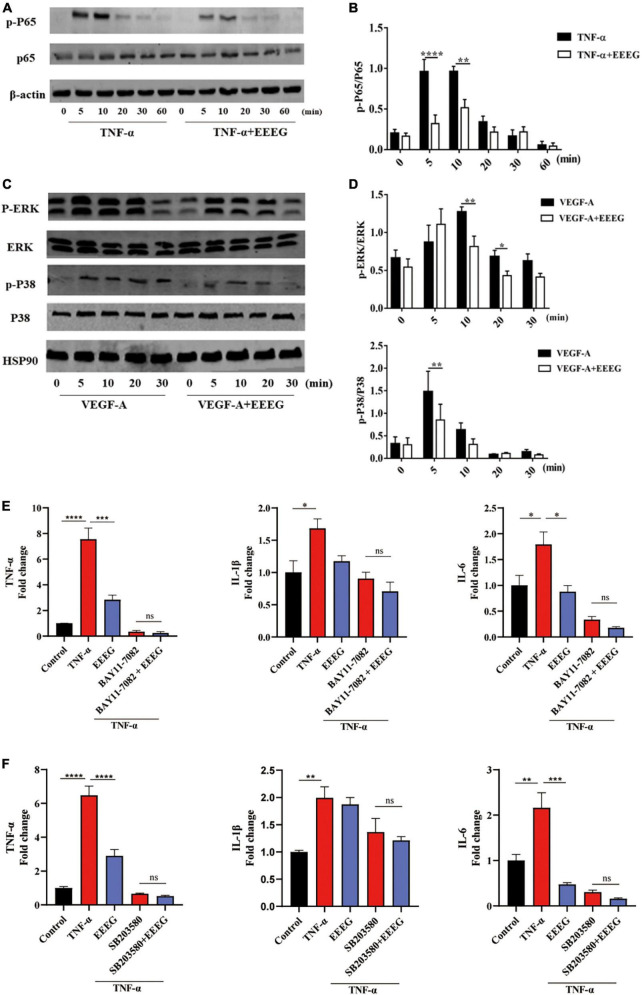
EG extract (EEEG) inhibits endothelial pro-inflammatory activation by targeting NF-κB and p38 MAPK signaling pathways. **(A,B)** Western blot analysis of the phosphorylation of p65 in HUVECs pretreated with or without EEEG (500 μg/mL) for 24 h followed by TNF-α (10 ng/ml) stimulation for 0, 5 10, 20, 30, and 60 min **(A)** and quantification of phosphorylated p65 normalized to total p65 (*n* = 3) **(B)**. **(C,D)** Western blot analysis of the phosphorylation of ERK and p38 MAPK in HUVECs pretreated with or without EEEG (500 μg/mL) for 24 h followed by stimulation with VEGF-A (150 ng/ml) for 0, 5 10, 20, and 30 min **(C)**; quantification of phosphorylated ERK and p38 MAPK normalized to total ERK and p38 MAPK (*n* = 3) **(D)**. **(E)** Gene expression levels of *Tnf*-α, *Il-1*β, and *Il-6* in HUVECs pretreated with or without EEEG (500 μg/mL) while in the presence or absence of NF-κB inhibitor BAY11-7082 (5 μM) for 24 h, followed by stimulation with or without TNF-α (10 ng/ml) for 4 h (*n* = 3). **(F)** Gene expression levels of *Tnf*-α, *Il-1*β, and *Il-6* in HUVECs pretreated with or without EEEG (500 μg/mL) while in the presence or absence of p38 MAPK inhibitor SB203580 (20 μM) for 24 h, followed by stimulation with or without TNF-α (10 ng/ml) for 4 h (*n* = 3). Data are mean ± SEM. *P < 0.05, **P < 0.01, ***P < 0.001, ****P < 0.0001.

NF-κB and MAPKs are the major signaling molecules that regulate endothelial inflammatory cascades (8, 12). To further investigate the mechanisms by which EEEG attenuates endothelial pro-inflammatory activation, we examined the effects of EEEG on activating these signaling pathways in HUVECs. In response to TNF-α stimulation, NF-κB p65 showed increased phosphorylation within 10 min. At the same time, EEEG-treated HUVECs exhibited a pronounced decrease in the phosphorylation of NF-κB p65 compared with vehicle controls (Figures 5A, B). Besides, VEGF-A is implicated in the induction of inflammatory response in addition to its well-known pro-angiogenic role (26, 27). We thus also used VEGF-A-stimulated HUVECs as a second model of endothelial inflammation besides TNF-α stimulation. Upon treatment of EEEG, the phosphorylation of ERK and p38 MAPK induced by VEGF-A was significantly reduced (Figures 5C, D). These data indicate that EEEG may attenuate endothelial pro-inflammatory activation by suppressing the activation of NF-κB and MAPK signaling pathways. To further validate our hypothesis, we investigated the effect of inhibiting NF-κB, ERK, and p38 MAPK signaling in TNF-α-induced HUVECs with their respective specific pharmacological inhibitors BAY11-7082, SD98059, and SB203580. We compared the expression of pro-inflammatory mediators *Tnf*-α, *Il-1*β, and *Il-6* in HUVECs at baseline, with TNF-α stimulation in the presence of EEEG, treatment with pharmacological inhibitors, or both. The expression of pro-inflammatory mediators *Tnf*-α, *Il-6* and to a lesser extent *Il-1*β, was markedly decreased in the presence of EEEG, BAY11-7082 or SB203580 compared to TNF-α-treated cells (Figures 5E, F). Notably, co-treatment of EEEG with BAY11-7082 or SB203580 showed that EEEG exerted no effect in further attenuating the expression of *Tnf*-α, *Il-1*β, and *Il-6* as compared with BAY11-7082 or SB203580 treatment alone, indicating no synergistic or additive effect on the inhibition of endothelial pro-inflammatory activation (Figures 5E, F). In contrast, co-treatment of EEEG with SD98059 exhibited a synergistic effect on the reduction of *Il-1*β expression level (Supplementary Figure 6). These data suggest that EEEG suppresses endothelial pro-inflammatory activation, at least in part, through NF-κB and p38 MAPK, but not ERK signaling pathway. Together with our *in vivo* findings, it is conceivable that inhibition of NF-κB- and p38 MAPK-mediated endothelial activation by EEEG contributes to the attenuated cardiac inflammatory response following MI and, thus, protects against MI-induced myocardial injury.

## Discussion

This study used a preclinical rat model of permanent LAD ligation-induced MI to explore whether EG has potential pharmacological effects on an ischemic cardiac injury. EEEG, the 30% ethanol fraction of EG extract, significantly attenuated myocardial infarct size, improved cardiac function, prevented adverse cardiac remodeling, and decreased cardiac inflammation after MI in rats. Mechanistically, we demonstrated that EEEG suppressed endothelial pro-inflammatory activation by inhibiting NF-κB, ERK, and p38 MAPK signaling pathways, which may subsequently counteract inflammatory damage to the ischemic heart. Our data provided the cellular and molecular basis through which EG exerts a cardioprotective effect in response to MI.

Experimental and clinical evidence has indicated a crucial role of inflammation in the pathogenesis of IHD (28). The recent immuno-suppressive Colchicine Cardiovascular Outcomes Trial (COLCOT) has shown encouraging results among patients with MI and strongly supported the inflammatory hypothesis of the pathogenesis of coronary artery disease (9, 10). Therefore, inflammation is now recognized as a treatment target for acute ischemic cardiovascular events (29). EG is a traditional Tibetan folk medicine that has long been widely used for health care. However, the direct pharmacological action of EG on IHD has yet to be investigated. The current study indicated that EEEG protects against MI injury induced by permanent LAD ligation (Figures 1, 2). The therapeutic benefit of EEEG lies in its ability to suppress cardiac inflammatory responses, as demonstrated by decreased infiltration of CD45^+^ leukocytes and CD68^+^ macrophages and reduced expression of pro-inflammatory mediators in the infarcted myocardium (Figures 3A–C). Significantly, attenuated myocardial inflammation mediated by EEEG facilitated the cardiac reparative process, characterized by increased CD31^+^ ECs after MI (Figures 3D, E). These findings suggest that EEEG may promote neovascularization and post-infarct healing, which is worth further investigation.

Ischemia leads to cardiac tissue damage and necrosis following MI, which initiates an acute pro-inflammatory response. Among cellular effectors of the inflammatory reaction in the ischemic heart, ECs play a fundamental role (8, 11). Danger signals released by dying cardiomyocytes induce rapid endothelial activation, manifesting upregulated expression of endothelial adhesion molecules as well as pro-inflammatory cytokines and chemokines, thereby promoting immune cell infiltration and complicating inflammation in the infarcted heart (30). In this study, using the TNF-α-stimulated HUVECs model of endothelial inflammation, we showed that EEEG significantly decreased the expression of adhesion molecule *Vcam-1* and pro-inflammatory cytokines *Tnf*-α, *Il-6*, and to a lesser extent, *Il-1*β, and inhibited monocyte-EC adhesion (Figures 4C–E). These results strongly indicate the inhibitory role of EEEG in endothelial pro-inflammatory activation. Since EEEG attenuated the infiltration of CD45+ leukocytes and CD68+ macrophages and reduced the production of inflammatory mediators in the ischemic myocardium *in vivo* (Figures 3A–C), it is reasoned that EEEG protects against MI-induced cardiac inflammation through a mechanism involving attenuation of endothelial pro-inflammatory activation.

NF-κB is a central intracellular signaling molecule involved in the induction of inflammatory responses (8). It has been known that the production of endothelial adhesion molecules and inflammatory cytokines is under the tight control of NF-κB (31). Upon inflammatory stimulation, NF-κB p65 becomes activated and binds to the promoter region of pro-inflammatory genes, triggering a series of inflammatory responses (31). Additionally, ERK and p38 MAPK also participate in response to inflammatory stimulation (32). Blockade of MAPK signaling activity suppresses endothelial pro-inflammatory activation, leading to the improvement of cellular damage (33). In the current study, we showed that the levels of phosphorylation of NF-κB p65, ERK, and p38 MAPK were markedly decreased in TNF-α- or VEGF-A-induced HUVECs as a result of EEEG pretreatment (Figures 5A–D).

Further mechanistic studies using pharmacological inhibitors demonstrated that EEEG specifically targets NF-κB and p38 MAPK signaling to suppress endothelial pro-inflammatory activation (Figures 5E, F). Moreover, previous studies have evidenced that both ERK and p38 MAPK positively regulates NF-κB activity in activated HUVECs, and inhibition of these MAPKs decreases NF-κB activation and immune disorders (34, 35). In this regard, it is reasoned that the reduced ERK and p38 MAPK activation after EEEG pretreatment in this study may also be involved in preventing NF-κB activation, eventually resulting in attenuated adhesion molecules and pro-inflammatory cytokine gene expression.

Although our data suggest that EEEG attenuates endothelial pro-inflammatory activation, we cannot exclude its pharmacological action on other cell types may also contribute to the reduced inflammatory response post-MI. Besides ECs, it has been shown that cardiomyocytes, neutrophils, monocytes/macrophages, mast cells, lymphocytes, and fibroblasts are critical cellular effectors of the inflammatory response after MI (8, 36). Thus, the role of EEEG in these cells in the context of MI-induced inflammation is worthy of further investigation. Furthermore, it has been reported that myocardial angiogenesis plays an essential role in cardiac repair and tissue remodeling. After MI, an adequately coordinated angiogenic response can be boosted to reduce scarring and adverse LV remodeling (37, 38). In this study, we observed that CD31^+^ cells were significantly increased in the hearts of MI rats treated with EEEG (Figures 3D, E). Consistently, HUVEC proliferation was also markedly accelerated by EEEG treatment *in vitro* (Figure 4B and Supplementary Figure 5). These observations imply that EEEG may promote myocardial angiogenesis and the post-infarct healing process, which potentially contributes to the protective effects of EEEG on MI injury. The primary regulators of angiogenesis include a series of signaling pathways such as PI3K/Akt, MAPK, Notch, JAK/STAT, Wnt/β-catenin, Hippo, and Sonic hedgehog (39). Previous studies have demonstrated that MAPK activation promotes angiogenesis (40, 41). In contrast, our data showed that EEEG attenuated ERK and p38 MAPK signaling in VEGF-A-induced HUVECs (Figures 5C, D), suggesting that the altered MAPK activity may not be responsible for EEEG-induced endothelial proliferation and angiogenesis. Thus, further studies are warranted to substantiate the role of EEEG in angiogenesis and explore the underlying molecular mechanism.

In summary, for the first time, the present study provides evidence that EEEG protects against ischemic cardiac injury and attenuates myocardial inflammation in response to MI *in vivo*. We propose a model in which EEEG inhibits NF-κB, ERK, and p38 MAPK signaling pathways, thereby suppressing endothelial pro-inflammatory activation and cardiac inflammatory response in the hearts following MI-induced injury. Our study provides an experimental basis for understanding the protective role of EG in MI and sheds light on the clinical use of EG in managing ischemic cardiovascular disease.

## Data availability statement

The original contributions presented in this study are included in the article/Supplementary material, further inquiries can be directed to the corresponding authors.

## Ethics statement

This animal study was reviewed and approved by the Institutional Animal Care and Use Committee of the Jiangxi University of Chinese Medicine.

## Author contributions

CZ and JY designed the study and coordinated all experimental work. DW, LT, XL, YZ, LS, SZ, and CZ conducted the experiments and collected the data. DW, LT, XL, YZ, LS, SZ, AT, MQ, LQ, CZ, and JY analyzed and interpreted the data. DW, LT, CZ, and JY wrote the manuscript with valuable input from all other authors. All authors approved the submitted version of the manuscript.
