# Supplementary material for: Edgeworthia gardneri (Wall.) Meisn. extract protects against myocardial infarction by inhibiting NF-κB-and MAPK-mediated endothelial inflammation
Source: Front Cardiovasc Med. 2022 Dec 20;9:1013013. doi: 10.3389/fcvm.2022.1013013 (PMC9808090; doi:10.3389/fcvm.2022.1013013)
Supplement: Supplementary file 1 [file Data_Sheet_1.docx]

*Edgeworthia gardneri* (Wall.) Meisn. extract protects against myocardial infarction by inhibiting NF-κB- and MAPK-mediated endothelial inflammation

**Dan Wei^1,#^, Le Tang^1,#^, Lingqing Su^1^, Sufen Zeng^1^, Ajdora Telushi^2^, Xiaoya Lang^1^, Yanli Zhang^3^, Manman Qin^1^, Liang Qiu^1^, Chao Zhong^1,^*, and Jun Yu^2,^***

**^1^Center for Translational Medicine, Jiangxi University of Chinese Medicine, Nanchang, Jiangxi, China**

**^2^Department of Cardiovascular Sciences and Center for Metabolic Disease Research, Lewis Katz School of Medicine, Temple University, Philadelphia, PA, USA**

**^3^The National Pharmaceutical Engineering Center for Solid Preparation in Chinese Herbal Medicine, Jiangxi University of Chinese Medicine, Nanchang, Jiangxi, China**

**^#^These authors contributed equally to this work.**

***Correspondence:**

Chao Zhong, PhD

Center for Translational Medicine, Jiangxi University of Chinese Medicine, Nanchang, Jiangxi, China

Or

Jun Yu, MD,

Department of Cardiovascular Sciences, Center for Metabolic Disease Research,

Lewis Katz School of Medicine, Temple University, Philadelphia, PA 19140, USA.

E-mail: jun.yu@temple.edu

**Supplementary Table 1:** Analysis of chemical constituents from EEEG by UPLC-Q-TOF-MS

| NO | Molecular Formula | Ionic Mode | Extraction Mass | Found At Mass | Error（ppm） | Retention Time (t_R_, min) | Fragmentation (m/z) | Identity |
| --- | --- | --- | --- | --- | --- | --- | --- | --- |
| 1 | C_15_H_10_O_7_ | [M+H]^+^ | 303.04993 | 303.04972 | -0.7 | 26.38 | 122.0355, 165.0188, 137.0233, 152.0104, 178.0234 | Quercetin |
| [2](http://www.baidu.com/link?url=XBgxXrWgdD3ul4mSwFRIApbHITHtHsqO-J70JFcsAWgD__pLWnx35TFQ869zvt7yw9_ePcI4_JsXOg8W4uDytxUH5ps_qlkHIocCBSBhcC3) | C_30_H_26_O_13_ | [M+H]^+^ | 595.14462 | 595.14457 | -0.1 | 27.99 | 287.0545, 309.0966, 147.0428 | Tiliroside |
| 3 | C_10_H_10_O_4_ | [M+H]^+^ | 195.06519 | 195.06512 | -0.3 | 11.93 | 150.0731, 177.0541, 123.0434, 118.0397 | Ferulic acid |
| 4 | C_7_H_6_O_5_ | [M+H]^+^ | 171.0288 | 171.0286 | -1.1 | 1.53 | 153.0174, 135.0070, 125.0226 | Gallic acid |
| [5](http://www.baidu.com/link?url=eISCTQ2lfSLeFgAzdQFVy1X_qpFmlD9Nl5DRokvcQGHDoNZ2ClSWNSoGw78Vb28pGHd0aGBEYZxBO97ICn9bqvGWFcQJLlKk_JupL4CGSC_) | C_15_H_14_O_6_ | [M+H]^+^ | 291.08631 | 291.08631 | 0 | 6.33 | 167.0381, 121.0274, 137.0233, 151.0392 | Catechin |
| 6 | C_7_H_6_O_3_ | [M+H]^+^ | 139.03897 | 139.03885 | -0.9 | 6.33 | 93.0324, 77.0393, 121.0264 | Salicylic acid |
| 7 | C_15_H_10_O_6_ | [M+H]^+^ | 287.05501 | 287.0552 | 0.6 | 28 | 165.0165, 121.0272, 137.0236, 153.0178 | Luteolin |
| 8 | C_8_H_6_O_6_ | [M+H]^+^ | 199.02371 | 199.01884 | -24.5 | 5.23 | 199.0636, 181.0066, 153.0136, 125.0213 | (2,4,5-trihydroxy-phenyl)-glyoxylic acid |
| 9 | C_10_H_8_O_3_ | [M+H]^+^ | 177.05462 | 177.05452 | -0.6 | 11.92 | 131.0515, 77.0392, 162.0295, 134.0357, 117.0333, 101.0367 | 4-Methylumbelliferone |

**Supplementary Table 2:** Sequences of qRT-PCR primers used in this study

|  | Genes | Primer sequences (5'-3') |
| --- | --- | --- |
| *In vivo* | *Tnf-α* | Forward: GTGATCGGTCCCAACAAGGA  Reverse: CTTGGTGGTTTGCTACGACG |
|  | *Il-1β* | Forward: TGACTCGTGGGATGATGACG  Reverse: CAGCTCACATGGGTCAGACA |
|  | *Il-6* | Forward: CCAGTTGCCTTCTTGGGACT  Reverse: CTGGTCTGTTGTGGGTGGTA |
|  | *Icam-1* | Forward: ACCACGGAGCCAATTTCTCA  Reverse: TCGAGCTTCAGGACCCTAGT |
|  | *Gapdh* | Forward: AGAAGGCTGGGGCTCATTTG  Reverse: AGGGGCCATCCACAGTCTTC |
| *In vitro* | *Tnf-α* | Forward: CCTCTCTCTAATCAGCCCTCTG  Reverse: GAGGACCTGGGAGTAGATGAG |
|  | *Vcam-1* | Forward: TTTGACAGGCTGGAGATAGACT  Reverse: TCAATGTGTAATTTAGCTCGGCA |
|  | *Il-6* | Forward: ACTCACCTCTTCAGAACGAATTG  Reverse: CCATCTTTGGAAGGTTCAGGTTG |
|  | *Il-1β* | Forward: ATGATGGCTTATTACAGTGGCAA  Reverse: GTCGGAGATTCGTAGCTGGA |
|  | *Gapdh* | Forward: AGAAGGCTGGGGCTCATTTG  Reverse: AGGGGCCATCCACAGTCTTC |

**Supplementary Figure S1**


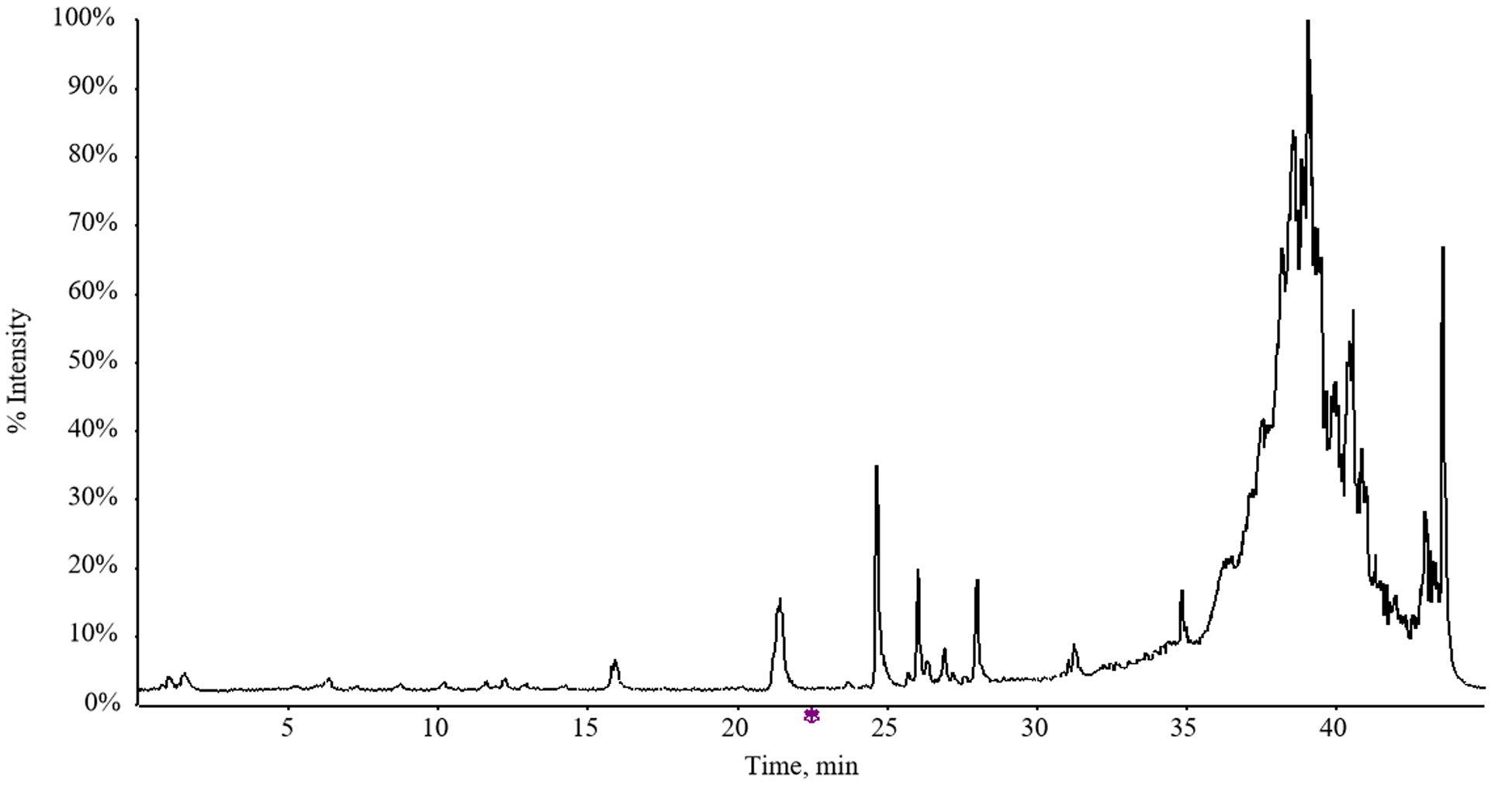


**Figure S1. The total ion chromatogram of EEEG by UPLC-Q-TOF-MS.**

**Supplementary Figure S2**

**
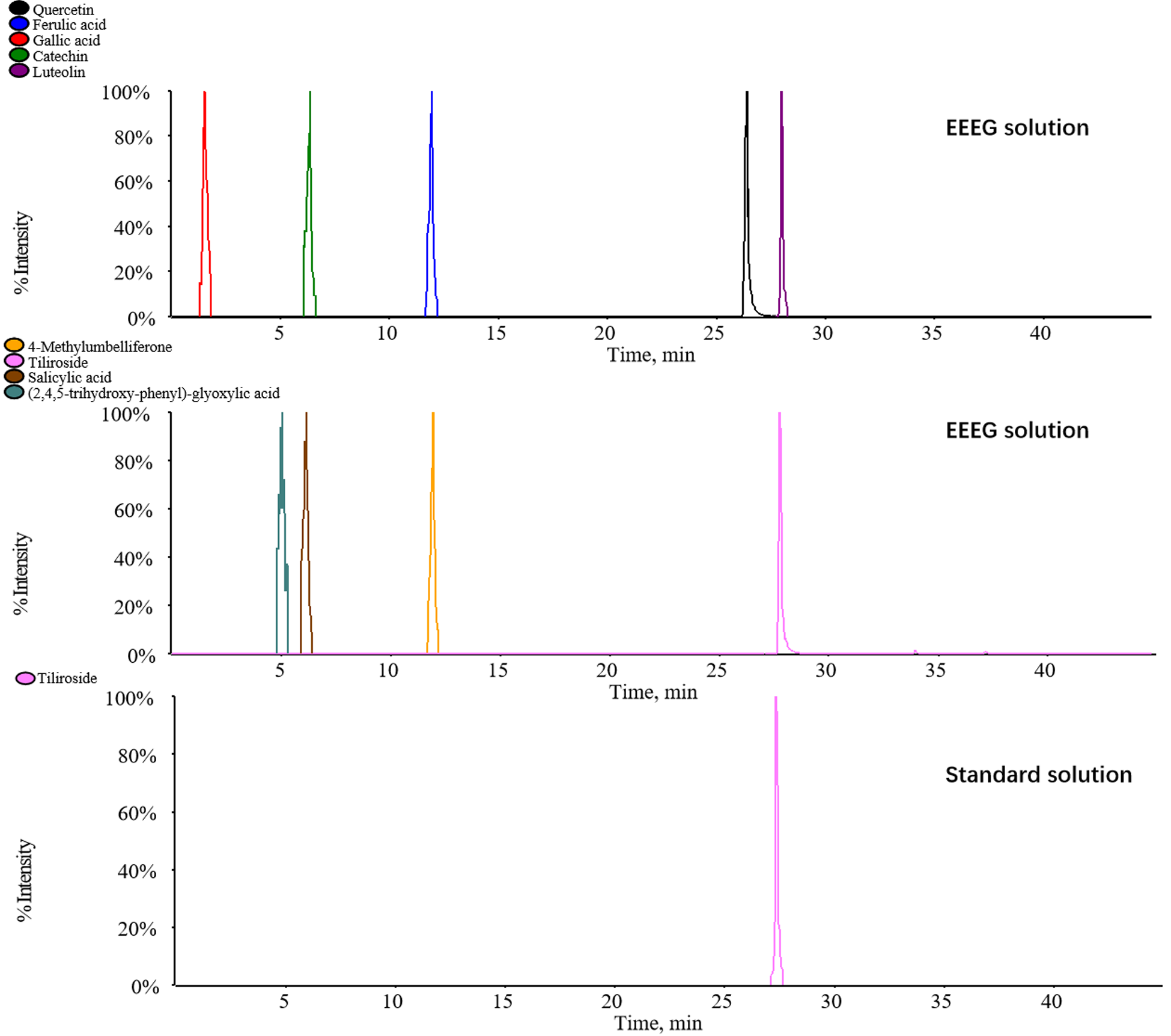
**

**Figure S2. Nine compounds were identified based on the total ion chromatograms of EEEG and the reference substance by UPLC-Q-TOF-MS.**

**Supplementary Figure S3**

**
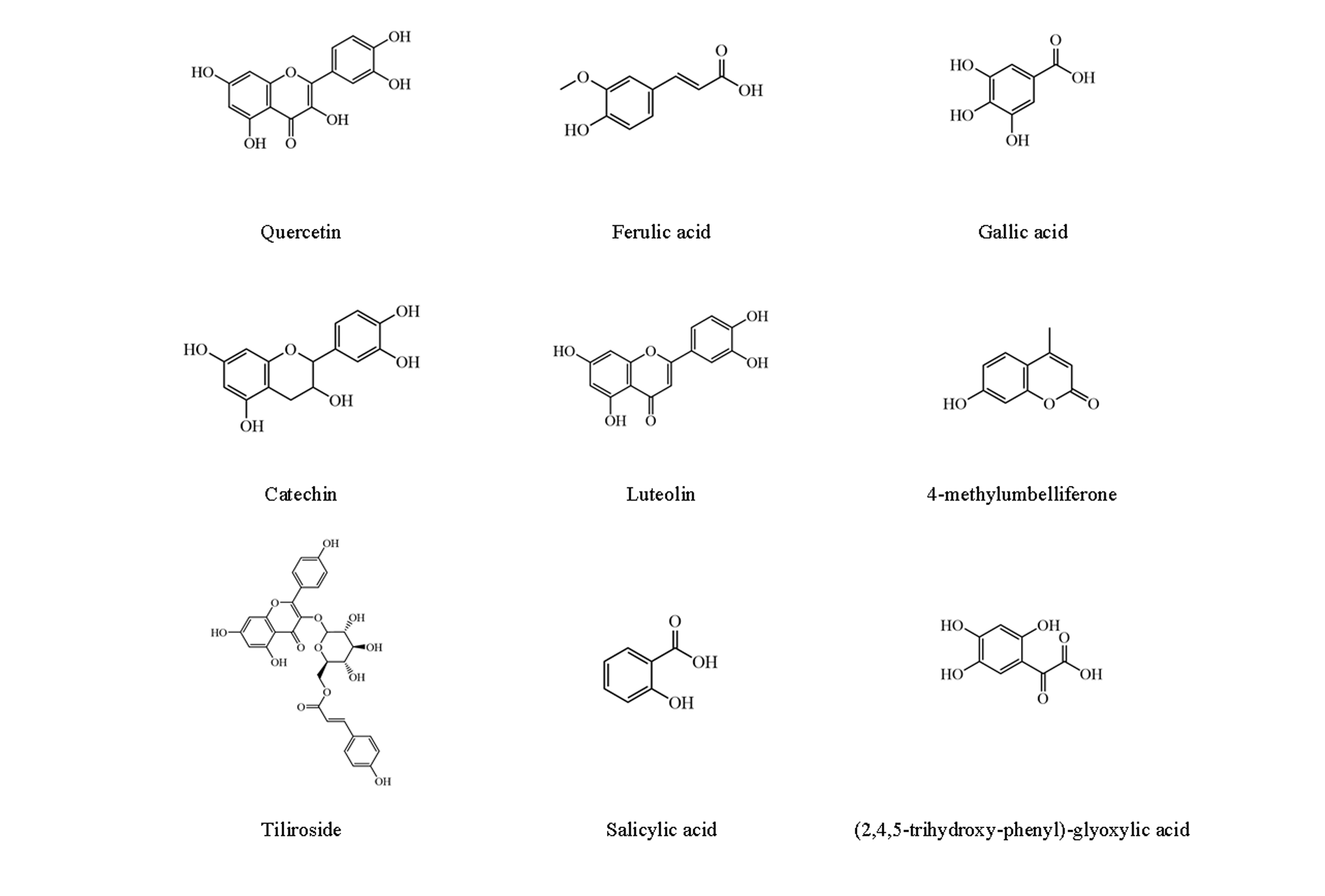
**

**Figure S3. The chemical structures of identified constituents in EEEG.**

**Supplementary Figure S4**


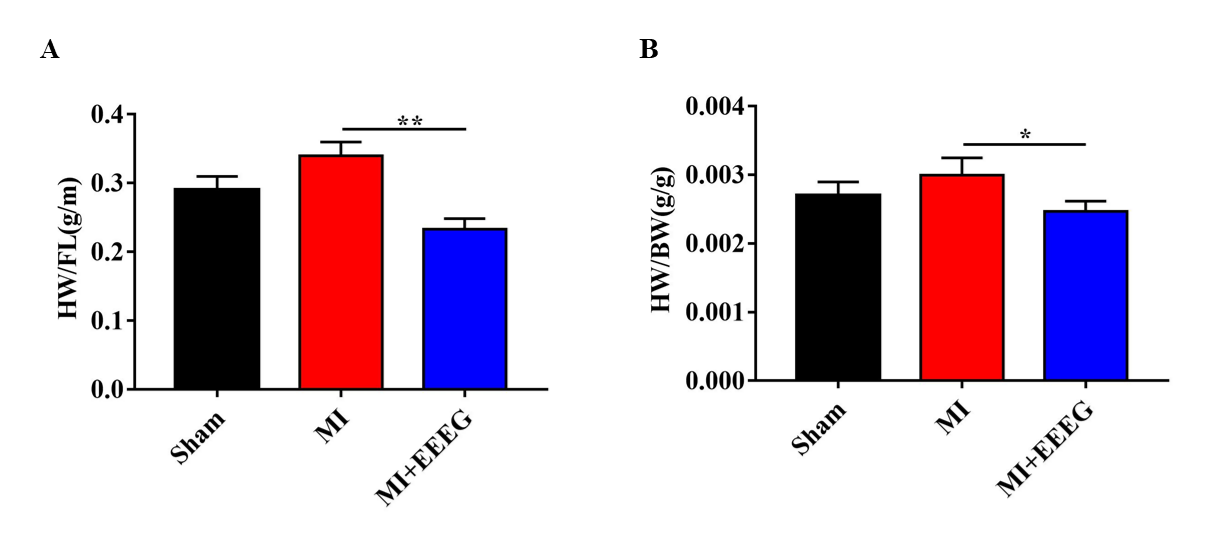


**Figure S4.** **EEEG attenuates MI-induced cardiac hypertrophy in rats.** **(A, B)** Heart weight-to-femur length **(A)** and heart weight-to-body weight **(B)** ratios were calculated in the vehicle and EEEG-treated rats 4 weeks after MI (n=8). Data are mean ± SEM. **P* < 0.05, ***P* < 0.01.

**Supplementary Figure S5**


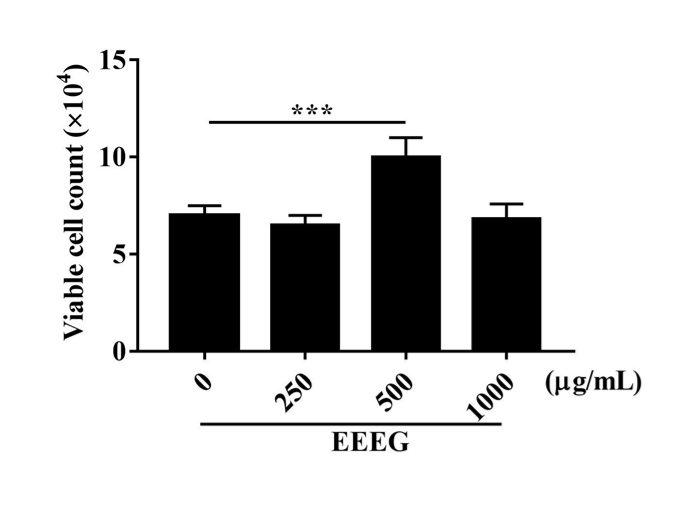


**Figure S5.** **EEEG promotes HUVECs proliferation.** HUVECs were incubated with indicated doses of EEEG for 48 h, and then cell proliferation was examined by direct cell counting (n=3). Data are mean ± SEM. ****P* < 0.001.

**Supplementary Figure S6**


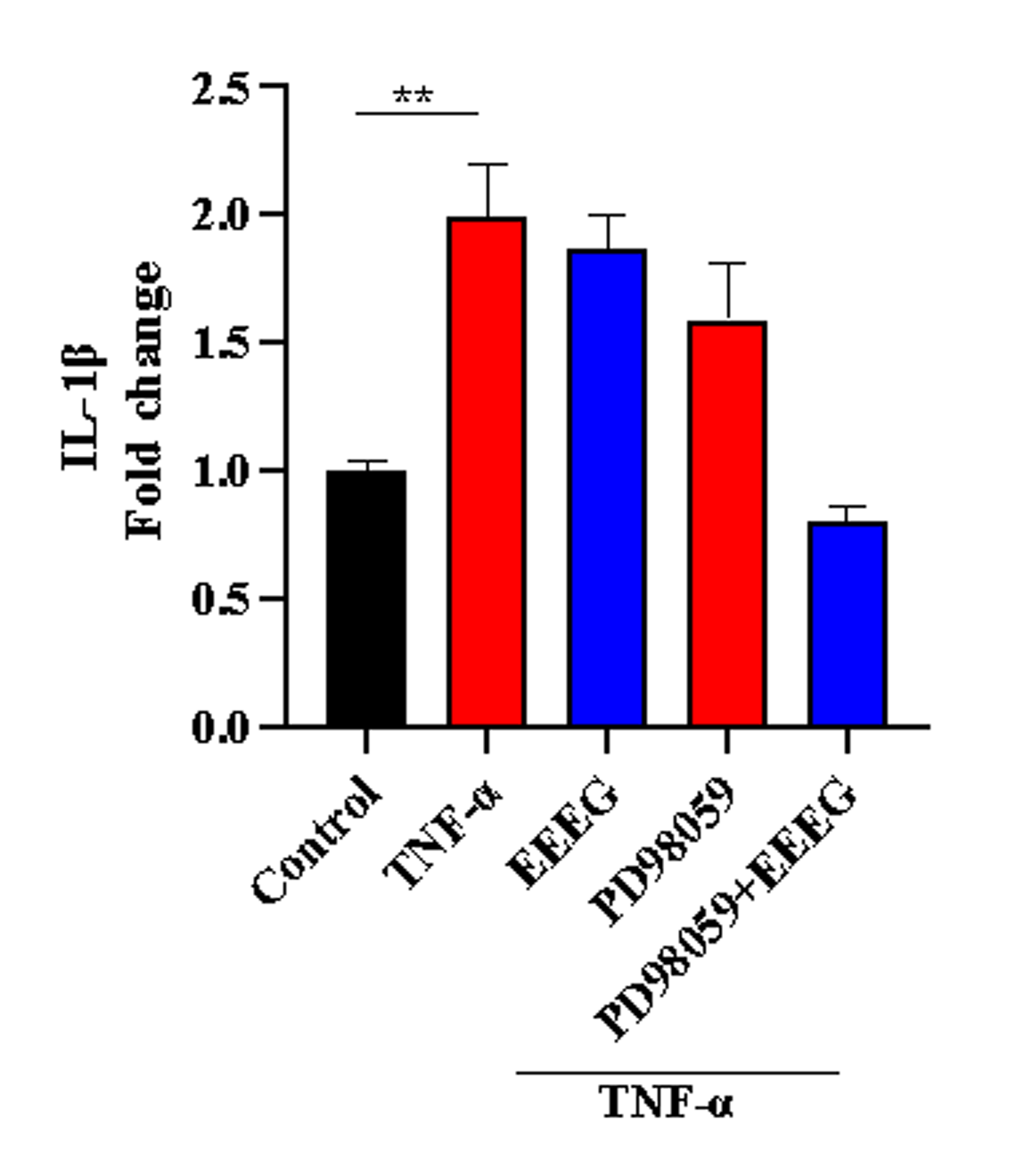


**Figure S6.** **EEEG attenuates endothelial inflammation in an ERK-independent manner.** Gene expression level of *Il-1β* in HUVECs pretreated with or without EEEG (500 μg/mL) while in the presence or absence of ERK inhibitor SD98059 (50 μM) for 24 h, followed by stimulation with or without TNF-α (10 ng/ml) for 4 h (n=3). Data are mean ± SEM. ***P* < 0.01.
